# Increased prescription rate of anti‐infective agents after diagnosis of myelodysplastic syndromes

**DOI:** 10.1002/jha2.422

**Published:** 2022-03-25

**Authors:** Johanne Rozema, Mels Hoogendoorn, Iris Potma, Inge ten Seldam, Nic J. G. M. Veeger, Robby E. Kibbelaar, Arjan A. van de Loosdrecht, Eric N. van Roon, the HemoBase Population Registry Consortium

**Affiliations:** ^1^ Unit of Pharmacotherapy Epidemiology and Economics Department of Pharmacy University of Groningen Groningen The Netherlands; ^2^ Department of Clinical Pharmacy and Pharmacology Medical Centre Leeuwarden Leeuwarden The Netherlands; ^3^ Department of Internal Medicine Medical Centre Leeuwarden Leeuwarden The Netherlands; ^4^ Science Bureau Medical Centre Leeuwarden Leeuwarden The Netherlands; ^5^ Department of Epidemiology University of Groningen University Medical Centre Groningen Groningen The Netherlands; ^6^ Pathology Friesland Leeuwarden The Netherlands; ^7^ Department of Haematology Amsterdam University Medical Centre Amsterdam The Netherlands

**Keywords:** anti‐infective agent, infections, myelodysplastic syndromes, population‐based

## Abstract

The a priori risk for infections in patients with myelodysplastic syndromes (MDS) is unknown. This study examines prescription rates of anti‐infective agents in MDS patients before and after diagnosis, in both in‐ and outpatient settings, to provide information on infection management in clinical practice.

We performed a population‐based study using the HemoBase registry, containing data of all MDS patients diagnosed since 2005 in Friesland, the Netherlands. Community and hospital pharmacies provided prescription data from 1995 to 2020.

Data were obtained for 203 of 292 patients (70%). Patients received significantly more anti‐infective agents, predominantly antibacterials (70%), after diagnosis compared to before: 148.7 defined daily dose/1000 days (DID) (95% CI: 146.9–150.5) and 55.1 DID (95% CI: 54.5–55.8, *p* < 0.01), respectively, corresponding to median 23.5 and 7.6 treatment days/year. Higher‐risk (449.9 DID) and lower‐risk patients (129.1 DID) both received significantly more anti‐infective agents after diagnosis; comorbidities, neutropenia, and age did not show significant differences relative to prescription rates. Before diagnosis, 10% of patients had infection‐related hospital admissions versus 38% after diagnosis.

In conclusion, MDS patients received significantly more anti‐infective agents compared to before diagnosis. This is the first study that has quantified the prescription rate of anti‐infective agents within and beyond the clinical setting in MDS.

## 1 INTRODUCTION

Myelodysplastic syndromes (MDS) are a group of clonal bone marrow disorders [1]. MDS is predominantly a disease of the elderly, with a median age at diagnosis of 75 years and a high prevalence of comorbidities [2, 3]. Many MDS patients suffer from neutropenia and/or neutrophil dysfunction; therefore the risk for infections is of particular interest [4, 5]. The best supportive care, such as treatment with erythropoiesis‐stimulating agents, granulocyte colony‐stimulating factors, or blood transfusions, is often aimed at improving anemia‐related symptoms. To the best of our knowledge, standard protocols for antimicrobial prophylaxis in MDS do not exist. The risk for infections is high, with infectious complications present in 15%–40% of patients in the studied populations [5, 6, 7, 8]. Moreover, infections are associated with increased mortality and account for the death of 20%–38% of MDS patients [8, 9, 10]. In addition, infections have a negative impact on quality of life and health care costs [10].

Current estimates of infection rates are based on results from clinical trials or retrospective analyses that only reviewed hospital records [5, 6, 7, 8, 11, 12]. Most studies that analyzed infectious complications focused on specific subgroups of the MDS population, such as specific treatment (e.g. hypomethylating agents [HMA] or a hematopoietic stem cell transplantation [HSCT]), or type of infection (e.g., invasive fungal infections) [13, 14, 15, 16, 17]. These studies have shown that certain MDS patients have a higher risk for infection; however, the prescription rate of anti‐infective agents in different MDS risk groups has not been investigated. Consequently, detailed information about the risk for infections translating into the prescription of anti‐infective agents for MDS in clinical practice is lacking. Furthermore, infections and prescriptions for anti‐infective agents in first‐line care have not been considered.

In contrast to the inpatient setting, outpatient care generally involves empirical treatment, as bacterial cultures are rarely available. One method of studying infections in an outpatient setting is to investigate the prescription of anti‐infective agents. This approach is particularly useful in examining the burden of possible infections [18, 19]. The HemoBase registry offers the means to assess the prescription of anti‐infective agents in MDS patients in Friesland, a province in the Netherlands with ±650,000 inhabitants. This registry includes all hemato‐oncological patients diagnosed since 2005 and provides population‐based data on diagnosis, treatment, and the day‐to‐day practice of MDS patients [2, 20]. Combined with first‐line care data, it offers a unique view on how these patients are treated.

This study investigates the prescription rate of anti‐infective agents in MDS patients (lower‐ and higher‐risk MDS) in an outpatient and inpatient setting; it assesses the prescription of anti‐infective agents over time before and after diagnosis of MDS and differences in prescription rates according to MDS risk groups, comorbidities, neutropenia, and age.

## 2 METHODS

A retrospective, population‐based study was performed using the HemoBase registry [2, 20, 21]. The local Medical Ethics Committee confirmed the execution of the study without the need for ethical review. All living patients provided informed consent in accordance with the Helsinki Declaration (2013 revision) and Dutch regulations.

All persons newly diagnosed with MDS between January 1, 2005 and December 31, 2017 were included in the study and followed through December 31, 2020 (also see Supplementary Data) [2]. Community pharmacies and (dispensing) general practitioners (GPs) provided information about the prescription of anti‐infective agents from 10 years before MDS diagnosis (i.e., from 1995 onwards, when available) to the end of follow‐up. The information systems of community pharmacies contain detailed, up‐to‐date information on prescribed, and over‐the‐counter medication, which guaranteed a complete overview of the prescriptions for anti‐infective agents without potential recall bias. Dutch pharmacies are by law obliged to keep records of all patient data for at least 20 years [22]. Many records date back even further, yielding rich data on prescriptions for anti‐infective agents (see Supplementary Data) [19]. In addition, The Netherlands has a strict policy for prescribing anti‐infective agents, and the use of these agents is much lower compared to other countries [23, 24]. Therefore, pharmacy records provide a valid estimate of the use of anti‐infective agents in MDS patients [19, 23]. These outpatient data were combined with HemoBase registry data containing information about hospital admissions, and inpatient prescriptions of anti‐infective agents from the time of diagnosis onwards and enriched with data on hospital admissions and inpatient prescriptions of anti‐infective agents before MDS diagnosis.

The prescription rate of anti‐infective agents was presented as prescriptions/year, days of treatment (DOT)/year, defined daily dose (DDD) per 1000 inhabitant days (DID) for total prescriptions and outpatient prescriptions, and DDD per 100 hospital days for inpatient prescriptions according to the anatomical therapeutic chemical classification (ATC)/DDD index of the World Health Organization [25]. An infection‐related hospital admission was defined as hospitalization due to infection or hospitalization during which an infectious complication occurred, based on documentation in hospital records. Prescriptions for anti‐infective agents were categorized as prophylaxis or active treatment according to the prescription information about dosage, treatment duration, and indication. Transplant recipients were censored from the date of transplantation.

Patients served as their own control; we compared the prescription rate of anti‐infective agents in patients with MDS to their prescription rate before diagnosis, with a maximum of 10 years prior to diagnosis. Complete case analysis was performed for DID calculations (i.e., only patients with data before and after diagnosis were considered). In‐ and outpatient data were combined and calculated with the total outpatient follow‐up before and after diagnosis to calculate the total DID. Descriptive statistics were used to describe patient characteristics. Day‐to‐day use of anti‐infective agents in all patients before versus after MDS diagnosis was graphically depicted. In this analysis, each day of follow‐up was assessed for the prescription of anti‐infective agents. More than one prescription of an anti‐infective agent on the same day in an individual patient was counted as a single prescription. Univariate analyses were performed to assess differences in the delta of prescription rates for anti‐infective agents before diagnosis compared to after diagnosis in patients according to different baseline parameters: IPSS‐R, CCI score, absolute neutrophil count (ANC), and age. In these Kruskal–Wallis tests, there was no a priori ranking of groups. Patients with a follow‐up <6 months were excluded from this analysis. Statistical analyses were performed using IBM SPSS version 24 and SAS version 9.4.

## 3 RESULTS

A total of 292 MDS patients were identified, and their pharmacists and (dispensing) GPs were asked to provide information. We received the data on prescribed anti‐infective agents for 203 patients (70%), which comprised 3868 outpatient and 466 inpatient prescriptions for anti‐infective agents. For the remaining 89 patients (30%), data were present but not made available by the GP and/or pharmacist for our study. The patient characteristics are listed in Table 1. There were no significant differences in age, gender, MDS characteristics, and comorbidity between included and missing patients. Fourteen patients were censored for transplantation (Table 1), five patients had unknown transplantation status and were therefore censored from date of diagnosis.

**TABLE 1:** Patient characteristics

	Included patients *N* (%)	Missing patients *N* (%)	*p*‐Value
Total	203 (100)	89 (100)	–
Male	141 (69.5)	62 (69.7)	0.97
Age (median [range])	75.7 y (30.1–92.0)	73.3 y (18.2–88.8)	0.12
MDS subtype			0.12
** **SLD	31 (15.3)	11 (12.4)	
** **MLD	32 (15.8)	9 (10.1)	
** **RS‐SLD	35 (17.2)	10 (11.2)	
** **RS‐MLD	20 (9.9)	10 (11.2)	
** **Del5q	6 (3.0)	0 (0)	
** **EB‐1	37 (18.2)	17 (19.1)	
** **EB‐2	20 (9.9)	19 (21.3)	
** **U	4 (2.0)	2 (2.2)	
** **n.o.s.	18 (8.9)	11 (12.4)	
IPSS‐R			0.15
** **Low risk	113 (55.7)	37 (41.6)	
** **Very low	16 (7.9)	3 (3.4)	
** **Low	68 (33.5)	20 (22.5)	
** **Intermediate	29 (14.3)	14 (15.7)	
** **High risk	24 (11.8)	15 (16.9)	
** **High	15 (7.4)	7 (7.9)	
** **Very high	9 (4.4)	8 (9.0)	
** **Unknown	66 (32.5)	37 (41.6)	
Neutropenia (×10^9^/L)^a^			0.17
** **<0.5	16 (7.9)	12 (13.5)	
** **0.5–1.0	26 (12.8)	14 (15.7)	
** **1.1–1.8	44 (21.7)	10 (11.2)	
** **>1.8	107 (52.7)	47 (52.8)	
** **Missing	10 (4.9)	6 (6.7)	
CCI score			0.69
** **0	63 (31.0)	28 (31.4)	
** **1	39 (19.2)	21 (23.6)	
** **2–3	50 (24.6)	22 (24.7)	
** **≥4	48 (23.6)	16 (18.0)	
** **Unknown	3 (1.5)	2 (2.2)	
Transplant	14 (6.9)	–	–
Treatment^b^		–	–
** **Hypomethylating agents	35 (17.2)		
** **Lenalidomide	7 (3.4)		
** **Chemotherapy	23 (11.3)		
Follow‐up (median [range])		–	–
** **Before diagnosis	10.0 y (0.1–10.0)		
** **After diagnosis	1.9 y (0.1–10.7)		

*Note*: Values are presented as numbers (percentage), unless stated otherwise.

Abbreviations: CCI, Charlson comorbidity index; EB, excess blasts; IPSS‐R, revised international prognostic scoring system; MDS, myelodysplastic syndromes; MLD, multilineage dysplasia; n.o.s., not otherwise specified; RS‐MLD, ring sideroblasts and multilineage dysplasia; RS‐SLD, ring sideroblasts and single lineage dysplasia; SLD, single lineage dysplasia; U, unclassifiable; y, years.

^a^ Neutropenia was more often seen in higher‐risk MDS patients: 56.5% of higher‐risk patients had ANC <1.8 compared to 37.6% of lower‐risk patients (*p* = 0.080).

^b^ A patient can have ≥1 treatment.

**FIGURE 1:**
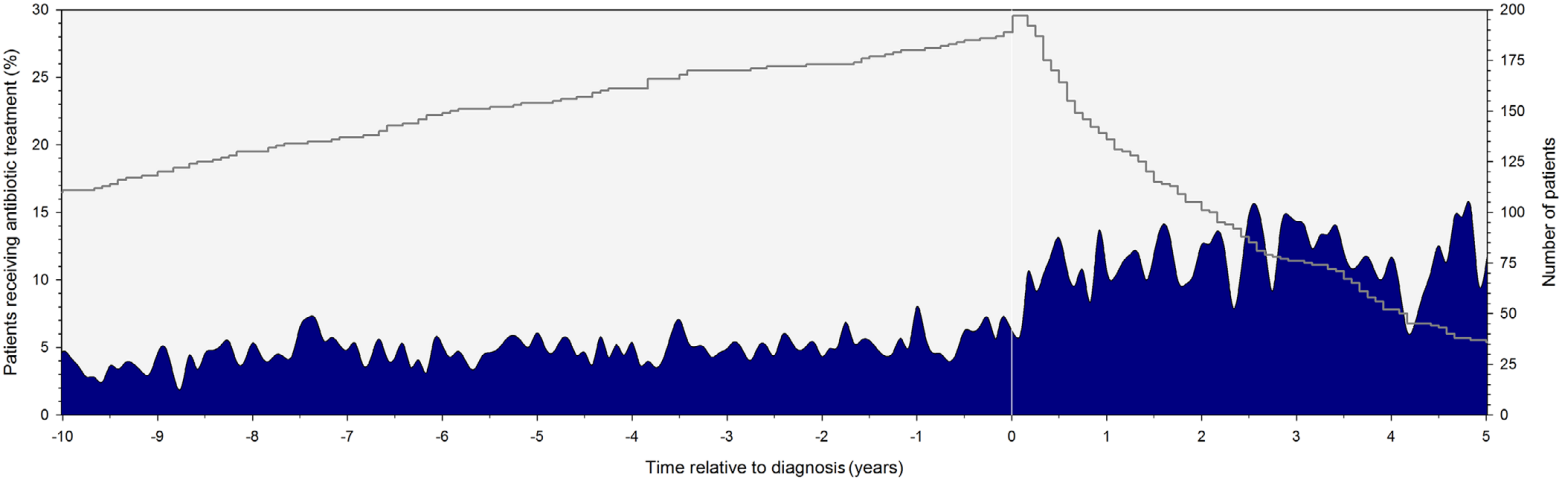
Average daily use per month of anti‐infective agents in myelodysplastic syndrome (MDS) patients over time. The left y‐axis represents the percentage of patients who receive anti‐infective treatment. The right y‐axis and grey line represent the number of patients in the database. Time = 0 is the moment of diagnosis.

The percentage of patients who received anti‐infective agents was relatively stable over the follow‐up period before MDS diagnosis was established, increased at the time of diagnosis, and remained at a stable level through the end of follow‐up (Figure 1). Patients received 55.1 DID (95% CI: 54.5–55.8) before diagnosis and 148.7 DID (95% CI: 146.9–150.5) after diagnosis (outpatient and inpatient data combined, *p* < 0.01).

**FIGURE 2:**
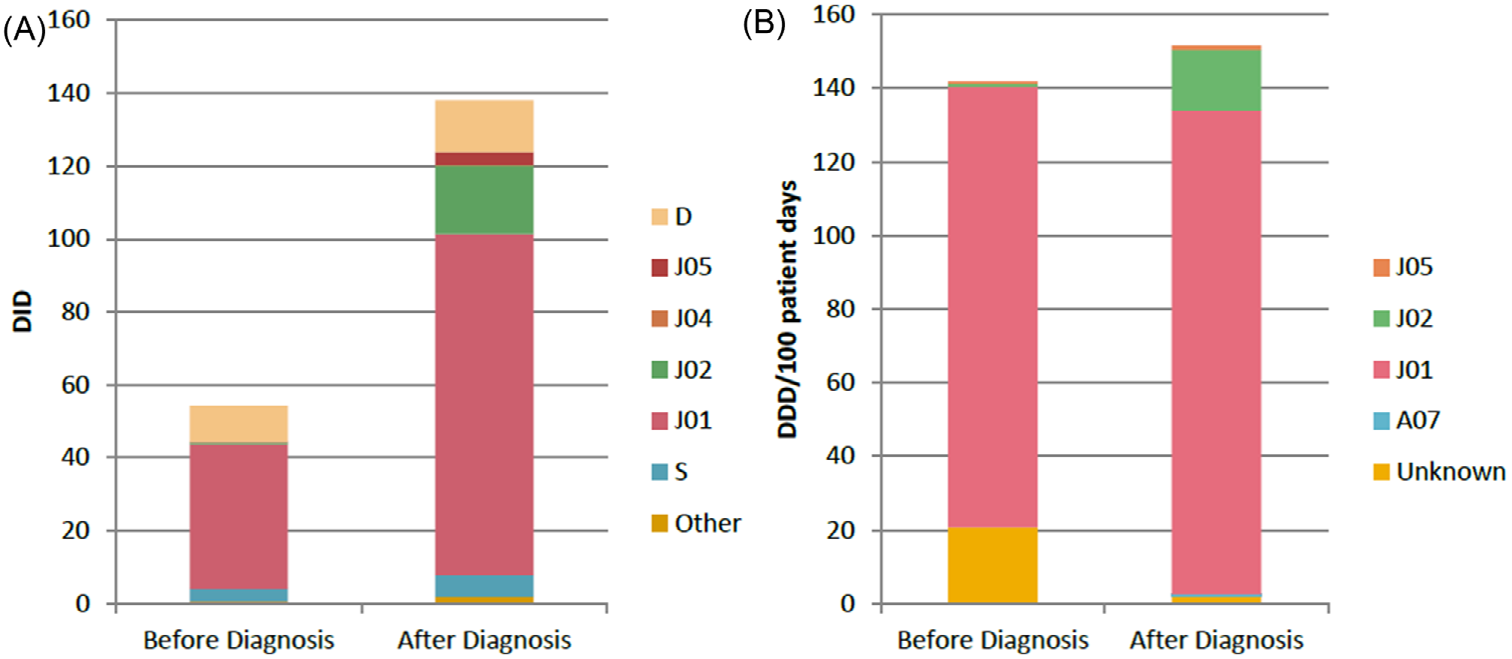
Prescription rate of anti‐infective agents in myelodysplastic syndrome (MDS) patients before and after diagnosis. (A) DID and types of anti‐infective agents in an outpatient setting. (B) DDD/100 patient days and types of anti‐infective agents in an inpatient setting. DDD, defined daily dose; DID, defined daily dose per 1000 inhabitant days. D, dermatologicals; J05, antivirals for systemic use; J04, antimycobacterials for systemic use; J02, antimycotics for systemic use; J01, antibacterials for systemic use; S, sensory organs; A07, alimentary tract and metabolism.

Patients received significantly more outpatient prescriptions/year after diagnosis (median 2.4; 95% CI: 2.1–3.0) compared to before diagnosis (median 0.8; 95% CI: 0.7–1.0; *p* < 0.01). The DOT/year significantly increased from a median of 7.6 (95% CI: 5.3–10.1) DOT/year before diagnosis to 23.5 (95% CI: 15.9–30.7) after diagnosis (*p* < 0.01). Patients received 53.8 DID (95% CI: 53.2–54.5) before diagnosis and 135.9 DID (95% CI: 134.2–137.7) after diagnosis (relative risk: 2.5, 95% CI: 2.5–2.6; *p* < 0.01). Antibacterial agents for systemic use (ATC‐group J01) were predominantly prescribed (Figure 2A). Overall, 17% and 35% of prescriptions concerned prophylactic use before and after diagnosis, respectively, including prophylaxis for treatment with HMA or chemotherapy. Prophylactic prescription predominantly involved treatment for recurring infections following an initial infection.

A total of 466 inpatient prescriptions for anti‐infective agents were used during 226 hospital admissions. There were 72 infection‐related hospital admissions before diagnosis and 154 after diagnosis. Before diagnosis, 20 patients (10%) had an infection‐related hospital admission during a median follow‐up of 10 years compared to 77 patients (38%) after diagnosis during a median follow‐up of 1.9 years. Twelve of these patients had an infection‐related hospital admission before and after diagnosis of MDS. Patients received 141.8 DDD/100 hospital days (95% CI: 132.6–151.6) before diagnosis compared to 151.6 DDD/100 hospital days (95% CI: 145.6–157.8) after diagnosis (relative risk: 1.1, 95% CI: 1.0–1.2; *p* = 0.09), indicating no differences in anti‐infective treatment once admitted to hospital. Patients mostly received antibacterial agents for systemic use (ATC‐group J01, Figure 2B). Following diagnosis, prophylactic anti‐infective agents during hospital admission (8% of prescriptions) were prescribed because of neutropenia or prophylactically after initial infection.

Univariate analyses were performed to assess differences in the delta of prescription rates for anti‐infective agents before diagnosis compared to after diagnosis among patients with differing IPSS‐R risk groups, CCI scores, ANCs, and ages. There was a statistically significant difference among IPSS‐R scores: higher‐risk MDS patients showed an increase of 5.95 weeks of treatment/year after diagnosis compared to before diagnosis versus 1.45 and 1.20 weeks/year for lower‐risk MDS patients and patients with an unknown IPSS‐R score, respectively (*p* = 0.029). Patients with a CCI score of 0–1 showed an increase of 1.1 weeks/year after diagnosis versus 2.7 weeks/year for those with a CCI score ≥2 (*p* = 0.12), and patients with an ANC ≤1 showed an increase of 2.8 weeks/year after diagnosis versus 1.2 weeks/year for those with an ANC > 1 (*p* = 0.12). Patients across the quartiles for age showed an increase of 1.4 weeks/year after diagnosis for Q1 (30–68 years), 3.3 for Q2 (68–75 years), 1.0 for Q3 (75–81 years), and 1.6 for Q4 (81–92 years) (*p* = 0.25). The DID, prescriptions/year, DOT/year, and hospital admissions for each subgroup are presented in Table 2.

**TABLE 2:** Prescriptions of anti‐infective agents according to IPSS‐R group

				Outpatient setting				Inpatient setting	
		DID (Median [95% CI])		Prescriptions/year (Median [95% CI])		DOT/year (Median [95% CI])		Hospital admissions *n* (%)	
	*N* (%)	Before MDS	After MDS	Before MDS	After MDS	Before MDS	After MDS	Before MDS	After MDS
Total	203	53.8 (53.2–54.5)	135.9 (134.2–137.7)	0.8 (0.7–1.0)	2.4 (2.1–3.0)	7.6 (5.3–10.1)	23.5 (15.9–30.7)	20 (9.9)	77 (37.9)
IPSS‐R risk group									
** **Low risk	113 (55.7)	64.8 (63.9–65.7)	129.1 (127.2–131.2)	0.7 (0.6–1.0)	2.1 (1.3–2.9)	5.4 (3.8–8.1)	15.5 (12.0–30.1)	5 (4.4)	42 (37.2)
** **High risk	24 (11.8)	35.7 (34.2–37.1)	449.9 (434.6–465.7)	0.4 (0.2–0.8)	3.9 (2.1–7.0)	3.3 (2.0–9.3)	36.5 (18.2–95.3)	4 (16.7)	13 (54.2)
** **Unknown	66 (32.5)	42.0 (41.0–43.0)	101.6 (98.6–104.7)	1.0 (0.8–1.4)	2.8 (2.3–5.1)	12.5 (9.5–14.5)	24.7 (16.8–37.5)	11 (16.7)	22 (33.3)
Age^a^									
** **≤75 years	104 (51.2)	33.6 (32.9–34.3)	139.3 (137.0–141.6)	0.7 (0.5–0.8)	2.2 (1.6–3.6)	5.5 (3.8–7.7)	22.3 (13.4–32.9)	11 (10.6)	40 (38.5)
** **> 75 years	99 (48.8)	74.8 (73.8–75.9)	131.3 (128.7–133.9)	1.0 (0.8–1.4)	2.5 (2.1–3.2)	10.5 (6.6–13.5)	23.5 (16.8–34.8)	9 (9.1)	37 (37.4)
CCI score									
** **0‐1	102 (50.2)	63.9 (62.8–64.9)	130.6 (128.4–132.9)	0.7 (0.5–1.0)	2.1 (1.4–2.9)	5.7 (4.2–9.3)	16.1 (12.8–25.4)	7 (6.9)	40 (39.2)
** **≥2	98 (48.3)	46.2 (45.4–47.0)	143.7 (141.0–146.4)	0.9 (0.7–1.2)	2.8 (2.2–4.4)	10.1 (5.9–11.9)	28.6 (18.3–41.7)	12 (12.2)	37 (37.8)
Neutropenia^a^									
** **≤1.0 × 10^9/L	42 (20.7)	40.0 (38.8–41.1)	209.7 (204.6–214.9)	0.8 (0.4–1.2)	3.5 (1.8–6.7)	5.9 (2.5–13.8)	33.0 (15.5–56.5)	5 (11.9)	19 (45.2)
** **>1.0 × 10^9/L	151 (74.4)	57.1 (56.3–57.9)	126.2 (124.3–128.1)	0.8 (0.7–1.1)	2.3 (1.9–2.9)	7.5 (5.1–9.6)	20.0 (15.2–29.3)	14 (9.3)	55 (36.4)

Abbreviations: CI, confidence interval; CCI, Charlson comorbidity index; DID, Defined daily dose (DDD) per 1000 inhabitant days; DOT, days of treatment; MDS, myelodysplastic syndromes; IPSS‐R, revised international prognostic scoring system.

^a^ At diagnosis.

**FIGURE 3:**
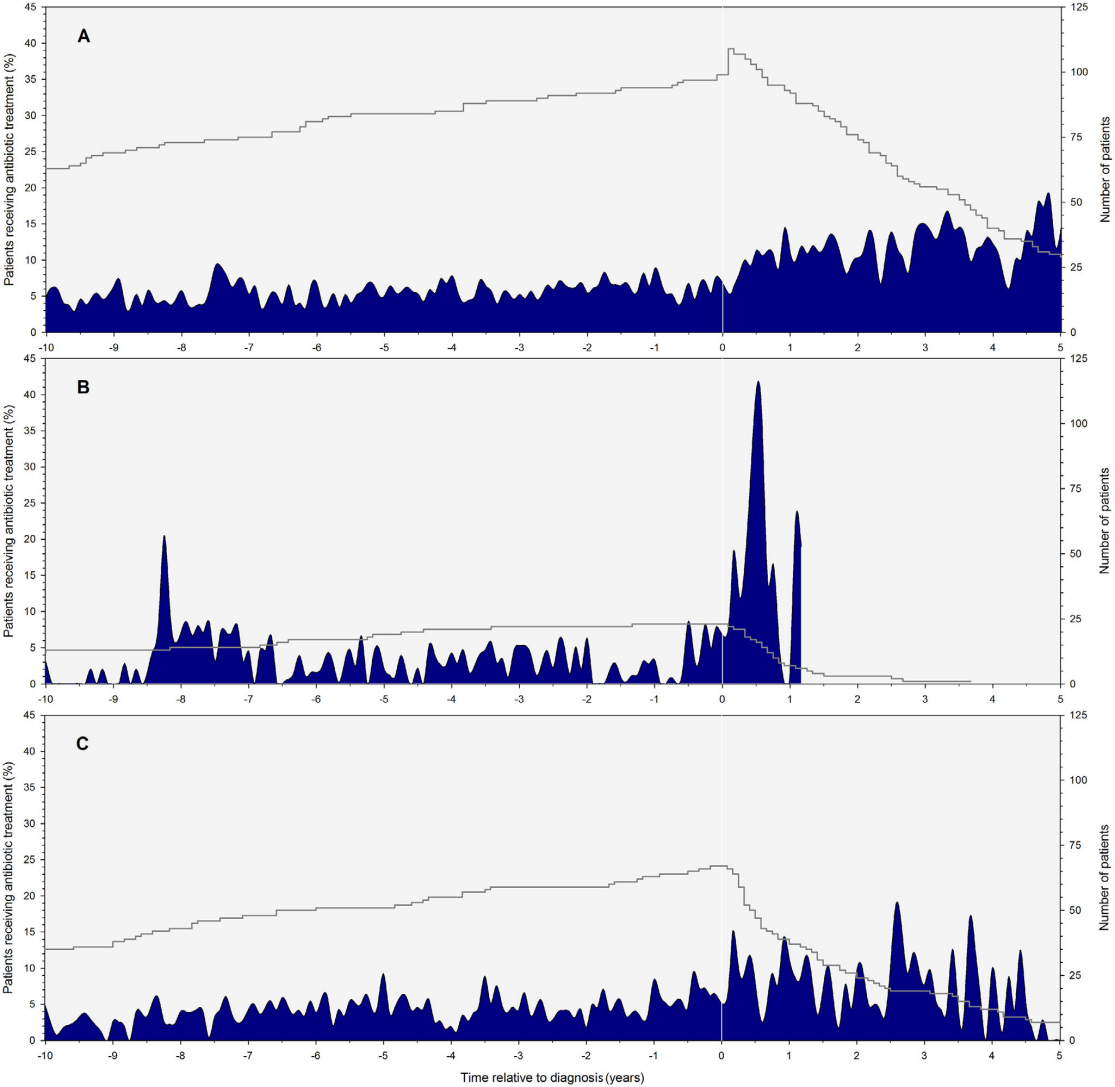
Average daily use per month of anti‐infective agents over time in myelodysplastic syndrome (MDS) patients from different revised international prognostic scoring system (IPSS‐R) risk categories. The left y‐axis represents the percentage of patients who receive anti‐infective treatment. The right y‐axis and grey line represent the number of patients in the database. Time = 0 is the moment of diagnosis. (**A)** Average daily use per month in lower‐risk MDS patients. (**B)** Average daily use per month in higher‐risk MDS patients. (**C)** Average daily use per month in MDS patients with unknown IPSS‐R score.

Both the lower‐ and higher‐risk MDS groups showed a relatively stable percentage of patients that received anti‐infective agents before diagnosis, which increased at the time of diagnosis until the end of follow‐up for both groups (Figure 3). Patients with higher‐risk MDS had a median of 3.9 prescriptions/year (95% CI: 2.1–7.0) compared to 2.1 prescriptions/year (95% CI: 1.3–2.9) for lower‐risk MDS patients (*p* = 0.28, Table 2). Higher‐risk patients also showed increased numbers of median DID, DOT/year, and infection‐related hospital admissions after diagnosis compared to patients with lower‐risk MDS (Table 2). To analyze whether intensive treatment was associated with an increased prescription rate of anti‐infective agents in higher‐risk patients, we performed additional DID calculations. Higher‐risk patients with intensive treatment received a median of 4.7 prescriptions/year of which 50% were prophylaxis, corresponding to 517.3 DID (95% CI: 499.1–536.0), after diagnosis compared to 2.6 prescriptions/year of which 12% were prophylaxis, corresponding to 156.0 DID (95% CI: 135.5–178.6), for higher‐risk patients without treatment (*p* < 0.01).

## 4 DISCUSSION

This study showed a significantly increased prescription rate of anti‐infective agents in patients after diagnosis of MDS as compared to before diagnosis. To our knowledge, this is the first descriptive population‐based study that provides an overall insight on prescribed anti‐infective agents within and beyond the clinical setting in a well‐defined representative MDS patient cohort. MDS patients had a 2.5 times higher chance of receiving anti‐infective agents in an outpatient setting after diagnosis. Higher‐risk MDS patients were particularly at risk for receiving anti‐infective agents; however, lower‐risk patients also showed a significant increase, as the prescription rate doubled following diagnosis. Most prescriptions were antibacterial agents and prescribed for the treatment of an active infection.

Our results showed that the percentage of patients who received anti‐infective agents was stable up to the time of diagnosis and increased at the time of diagnosis. This increase persisted through the end of follow‐up, indicating that anti‐infective agents were prescribed during the entire disease duration and did not decrease with time. This finding aligns with our expectations, as there is, besides HSCT, no cure for these patients. In addition, the prescriptions/year and DOT/year significantly increased by a factor of three since diagnosis, meaning patients had more than two prescriptions for anti‐infective agents per year and more than 3 weeks of treatment per year, a clinically significant increase compared to before diagnosis.

These results support the findings of previous research and observations that MDS patients are at increased risk for infections after diagnosis [5, 7, 10]. The rate of infection‐related hospital admissions agreed with earlier findings, as studies have shown infection‐related hospital admissions are common among MDS patients, with rates of 15%–61% during study periods of 1–4 years [7, 10, 14, 17, 26, 27].

In particular, higher‐risk MDS patients showed an increased risk for receiving outpatient anti‐infective agents after diagnosis. This may be explained by the severity of the disease, as this group more often has severe neutropenia, impaired function of (regulatory) T cells, a higher transfusion burden, and more interventions compared to lower‐risk patients and are therefore prone to infections [27, 28, 29]. Of note is the relatively short follow‐up of higher‐risk patients as shown in Figure 3, which can be explained by their poor prognosis, censoring for transplantation, and overall survival [2]. Although higher‐risk patients are predominantly treated with HMA or chemotherapy to restore bone marrow function, especially after the start of treatment, they are at increased risk for infections [5, 27, 28]. Indeed, there was an increased prescription rate for higher‐risk MDS patients who received treatment. However, lower‐risk patients also showed a significant increase in prescription rate: the DID doubled following MDS diagnosis. The effect of treatment should be further investigated in future studies, as this topic was beyond the scope of this study.

This study found that patients received 55.1 DID before MDS diagnosis, which is higher than prescription rates presented in other studies [23, 30, 31, 32, 33, 34]. The average total rate in 2019 was 9.5 DID in the Netherlands and 19.4 DID in Europe [23]. However, These data only included anti‐infective agents from ATC‐group J01, which may explain the lower figures compared to our data [23]. As 70% of our prescriptions consisted of agents from ATC‐group J01, our study still shows an increased rate (42.4 DID) compared to the literature. Our results suggest that MDS patients have an increased prescription rate before diagnosis compared to other patient groups, and the prescription rate further increases after diagnosis of MDS. Interestingly, the prescription rate was relatively stable over the 10 years leading up to diagnosis. Two studies have described the prescription of anti‐infective agents in nursing homes: one reported a DID of 44.8, the other described rates varying from 20 to 120 DID across European countries [32, 35]. Our results are in accordance with these findings, which included elderly populations such as ours. Studies describing prescription rates in other haemato‐oncological patients were, to our knowledge, not available. Hence, it remains difficult to assess whether MDS patients have an increased prescription rate of anti‐infective agents a priori, and further research is needed.

There are several possible explanations for the increased prescription rate of anti‐infective agents after diagnosis of MDS and the increased susceptibility to infections in this patient population. The first explanation is a functional impairment on a cellular level [36, 37, 38]. Bento et al. studied the monocytes of MDS patients and found that abnormalities may be associated with an impaired immune system [36]. Secondly, several studies have identified neutropenia or neutrophil dysfunction as risk factors for infection [4, 15, 27, 28, 39, 40]. Neutropenia was a common finding (42%) in this population and is not limited to a specific subtype of MDS, which supports our finding that the entire MDS population showed an increased prescription rate of anti‐infective agents [4, 28]. Finally, comorbidities may also contribute to infections in MDS [15, 28]. Two‐thirds of our study population had at least one comorbidity, which further supports the increased prescription rate of anti‐infective agents in these patients. Although not statistically significant, patients with a CCI score ≥2 indeed had a greater increase in prescription rate than patients with a CCI < 2. In larger study populations, the effect of comorbidities might be more visible. Antibacterial agents were most commonly prescribed, which agrees with earlier findings that MDS patients predominantly suffer from bacterial infections [28, 40, 41].

Regarding the study's strengths and weaknesses, one notable strength of this study is the large amount of detailed information we collected in an unselected MDS population. We had the opportunity to compare prescription rates of anti‐infective agents before and after diagnosis of MDS based on comprehensive prescription data from Dutch pharmacies. The detailed, up‐to‐date pharmacy information systems provided a complete overview without potential recall bias. Moreover, Dutch policies on prescribing anti‐infective agents are strict and therefore may act as an accurate reflection of infection rates [23, 24]. In addition, we received the data of 70% of the MDS population, and our study population consisted of a representative cohort of MDS patients [3, 42, 43, 44]. This investigation is the first population‐based study able to quantify the prescription rate of anti‐infective agents in MDS patients in outpatient and inpatient care. It can provide guidance for future investigations and the management of risk for infections in this patient population.

A limitation of this study is the age difference in the pre‐post MDS diagnosis comparison. Due to this design, the estimates of prescriptions of anti‐infective agents after MDS diagnosis were based on an older population. To limit this effect, a maximum follow‐up was set to 10 years before diagnosis. Furthermore, age at the time of diagnosis was not associated with increased prescription rates. Because of this design, characteristics such as gender and medical history were equal before and after diagnosis. Therefore, we feel this study shows an accurate comparison of the real‐world prescription rate of anti‐infective agents before and after diagnosis of MDS. Another limitation was that not all information could be retrieved for all patients, as this was a retrospective study, and it is possible that some inpatient data might have been missed. In addition, it was not feasible to study the effect of factors like neutropenia or comorbidities over time. These parameters should ideally be collected prospectively to avoid selection bias and confounding by indication. These data were unavailable in our dataset and should be further investigated. Still, this study encompassed many detailed data on MDS patients and their prescription rates over time, and we feel it provides important information on the management of MDS in clinical practice.

In conclusion, this study showed a significantly increased prescription rate of anti‐infective agents after diagnosis of MDS compared to before diagnosis. Higher‐risk patients were particularly at risk for receiving anti‐infective agents; however, lower‐risk patients also showed a significantly increased risk, as the prescription rate doubled after MDS diagnosis. To our knowledge, this is the first population‐based study that has quantified the prescription rate of anti‐infective agents within and beyond the clinical setting in a well‐defined representative MDS patient cohort. Further research on infectious complications in this patient group and the rational use of anti‐infective agents in a real‐world setting is warranted.

## CONFLICT OF INTEREST

The authors declare no conflict of interest.

## AUTHOR CONTRIBUTIONS

Johanne Rozema, Iris Potma, Eric N. van Roon, Mels Hoogendoorn, and Robby E. Kibbelaar designed the project. Johanne Rozema, Iris Potma, and Inge ten Seldam collected the data. Johanne Rozema wrote the manuscript. Johanne Rozema, Iris Potma, Inge ten Seldam, and Nic J.G.M. Veeger analyzed the data. Eric N. van Roon, Mels Hoogendoorn, Robby E. Kibbelaar, Arjan A. van de Loosdrecht, and Nic J.G.M. Veeger provided input in data analysis. All authors contributed to critical revision and gave final approval of the manuscript.

## Supporting information

Supplementary data
