# Supplementary material for: Increased prescription rate of anti‐infective agents after diagnosis of myelodysplastic syndromes
Source: EJHaem. 2022 Mar 25;3(3):775–84. doi: 10.1002/jha2.422 (PMC9422012; doi:10.1002/jha2.422)
Supplement: Supplementary file 1 — Supplementary data [file JHA2-3-775-s001.docx]

# Supplementary data

# Increased prescription rate of anti-infective agents after diagnosis of myelodysplastic syndromes

Johanne Rozema^1,2^, Mels Hoogendoorn^3^, Iris Potma^1^, Inge ten Seldam^1^, Nic J.G.M. Veeger^4,5^, Robby E. Kibbelaar^6^, Arjan A. van de Loosdrecht^7^, and Eric N. van Roon^1,2^, on behalf of the HemoBase Population Registry Consortium

^1^Unit of Pharmacotherapy, Epidemiology and Economics, Department of Pharmacy, University of Groningen, Groningen, the Netherlands;
^2^Department of Clinical Pharmacy & Pharmacology, Medical Centre Leeuwarden, Leeuwarden, the Netherlands;

^3^Department of Internal Medicine, Medical Centre Leeuwarden, Leeuwarden, the Netherlands;

^4^Science Bureau, Medical Centre Leeuwarden, Leeuwarden, the Netherlands;

^5^Department of Epidemiology, University of Groningen, University Medical Centre Groningen, Groningen, the Netherlands;

^6^Pathology Friesland, Leeuwarden, the Netherlands;

^7^Department of Hematology, Amsterdam University Medical Centre, Location VUmc, Amsterdam, the Netherlands;

A list of the HemoBase Population Registry Consortium appears in the acknowledgements.

**Author for correspondence**

Johanne Rozema, MSc
University of Groningen / Medical Centre Leeuwarden, Department of Clinical Pharmacy & Pharmacology
P.O. Box 888, 8901 BR Leeuwarden,
The Netherlands
Tel: +31 58 286 3483
Fax: +31 58 286 6606
Email: [hanne.rozema@rug.nl](mailto:hanne.rozema@rug.nl)
ORCID: 0000-0002-9454-8637

## **Supplementary methods**

**Study procedures**

The diagnosis of each patient was blindly revised according to the World Health Organization 2016 classification.^1-3^ The revision was performed by an expert panel consisting of a haematopathologist, hematologist, bone marrow cytologist, and clinical laboratory geneticist.^3^ Discrepancies were resolved by discussion until a consensus was reached.

**Definitions**

Revised International Prognostic Scoring System (IPSS-R) risk groups very low, low, and intermediate were defined as lower-risk MDS, whereas high and very high constituted higher-risk MDS.^4,5^ The IPSS-R could not be determined for all patients due to missing cytogenetic data or unsuccessful bone marrow aspirates. Such patients were analysed as a separate group. Comorbidities were scored according to the Charlson Comorbidity Index (CCI, without age adjustment).^6^ For statistical analyses, the IPSS-R and CCI scores at baseline were taken into consideration. Intensive treatment was defined as treatment with hypomethylating agents, lenalidomide, and/or chemotherapy.

**Prescriptions of anti-infective agents**

In this study, we compared the prescription rate of anti-infective agents before and after diagnosis of MDS. We received extensive prescription data from Dutch pharmacies and general practitioners and each prescription was carefully considered. The information systems of community pharmacies contain detailed, up-to-date information on prescribed and over-the-counter medication that a patient received as part of first-line care for the purpose of medication surveillance and reimbursement, which guaranteed a complete overview of the prescriptions for anti-infective agents without potential recall bias. If a patient moved or changed pharmacy, the data of all prescriptions from his pharmacy remain available for analysis. If a patient moved or changed pharmacy outside of Friesland, this pharmacy was not taken into account for the analysis. However, in Friesland, patients rarely move outside of the province. Dutch pharmacies maintain a strict record of all dispensed medications in their information system and are by law obliged to keep records of all patient data for at least 20 years.^7^ Many records date back even further, as pharmacies tend to keep these records to optimize patient care, yielding rich data on prescriptions for anti-infective agents.^8^ This detailed data enabled us to distinguish between prophylactic or empirical use of anti-infective agents. For all individual patients, the exact date of start and end of follow-up was known, meaning we were able to distinguish between ‘no use’ and ‘unknown use due to missing data’. In addition, the Netherlands has a strict policy for prescribing anti-infective agents, and the use of these agents is much lower in the Netherlands compared to other countries, such as Greece, France, or Italy.^9,10^ It is therefore rational and valid to consider pharmacy records when estimating the use of anti-infective agents in MDS patients.^8,9^ Outpatient data were combined with inpatient data from the HemoBase registry based in Friesland, the Netherlands.

## **References**

1. Arber DA, Orazi A, Hasserjian R, et al. The 2016 revision to the World Health Organization classification of myeloid neoplasms and acute leukemia. *Blood.* 2016;**127**(20):2391-2405.

2. Rozema J, Slim CL, Veeger NJGM, et al. A clinical effect of disease-modifying treatment on alloimmunisation in transfused patients with myelodysplastic syndromes: data from a population-based study. *Blood Transfus.* 2020;.

3. Rozema J, Hoogendoorn M, Kibbelaar R, van den Berg E, Veeger N, van Roon E. Comorbidities and malignancies negatively affect survival in myelodysplastic syndromes: a population-based study. *Blood Adv.* 2021;**5**(5):1344-1351.

4. Greenberg PL, Tuechler H, Schanz J, et al. Revised international prognostic scoring system for myelodysplastic syndromes. *Blood.* 2012;**120**(12):2454-2465.

5. Stauder R, Yu G, Koinig KA, et al. Health-related quality of life in lower-risk MDS patients compared with age- and sex-matched reference populations: a European LeukemiaNet study. *Leukemia.* 2018;**32**(6):1380-1392.

6. Charlson ME, Pompei P, Ales KL, MacKenzie CR. A new method of classifying prognostic comorbidity in longitudinal studies: development and validation. *J Chronic Dis.* 1987;**40**(5):373-383.

7. Wetten.nl. Afd 5. De overeenkomst inzake geneeskundige behandeling (WGBO), art. 454. *Burgerlijk wetboek Boek 7* 2020.

8. de Jong LAW, van der Linden PD, Roukens MMB, et al. Consecutive antibiotic use in the outpatient setting: an extensive, longitudinal descriptive analysis of antibiotic dispensing data in the Netherlands. *BMC Infect Dis.* 2019;**19**(1):84-019-3732-x.

9. European Centre for Disease Prevention and Control. Antimicrobial consumption in the EU/EEA - Annual Epidemiological Report for 2019. Stockholm: 2020. Report No.: 2019.

10. de Greeff SC, Schoffelen AF, Verduin CM. NethMap 2020 - Consumption of antimicrobial agents andantimicrobial resistanceamong medically important bacteriain the Netherlandsin 2019. *RIVM.* 2020;(RIVM report 2020-0065).
